# Elevated circulating tumor cells reflect high proliferation and genomic complexity in multiple myeloma

**DOI:** 10.1002/hem3.70218

**Published:** 2025-09-23

**Authors:** Juan‐Jose Garces, Benjamin Diamond, Tereza Sevcikova, Serafim Nenarokov, Daniel Bilek, Eva Radova, Ondrej Venglar, Veronika Kapustova, Ross Firestone, Kylee Maclachlan, Anish Simhal, Lucie Broskevicova, Jan Vrana, Ludmila Muronova, Tereza Popkova, Jana Mihalyova, Hana Plonkova, Michael Durante, Bachisio Ziccheddu, Michal Simicek, Hearn Jay Cho, George Mulligan, Jonathan Keats, David Zihala, Ola Landgren, Roman Hajek, Saad Usmani, Francesco Maura, Tomas Jelinek

**Affiliations:** ^1^ Myeloma Service, Department of Medicine Memorial Sloan Kettering Cancer Center New York New York USA; ^2^ Sylvester Comprehensive Cancer Center, Sylvester Myeloma Institute University of Miami Miami Florida USA; ^3^ Department of Hematooncology Faculty of Medicine University of Ostrava Ostrava Czech Republic; ^4^ Department of Hematooncology University Hospital Ostrava Ostrava Czech Republic; ^5^ Department of Biology and Ecology Faculty of Science University of Ostrava Ostrava Czech Republic; ^6^ Department of Medical Physics Memorial Sloan Kettering Cancer Center New York New York USA; ^7^ Icahn School of Medicine at Mount Sinai, Tisch Cancer Institute New York City New York USA; ^8^ Multiple Myeloma Research Foundation Norwalk Connecticut USA; ^9^ Integrated Cancer Genomics Division, Translational Genomics Research Institute Phoenix Arizona USA

## Abstract

Circulating tumor cells (CTCs) have emerged as a key prognostic factor in newly diagnosed multiple myeloma (NDMM). However, it remains unclear if high CTC counts represent a mere surrogate of tumor burden or might reflect a distinct genomic or transcriptomic entity. In this study, we characterized the genomic and transcriptomic features associated with CTC burden and assessed their combined prognostic value in NDMM patients. We analyzed 540 NDMM patients from the CoMMpass dataset with available baseline CTC information and matched bone marrow transcriptomic (*n* = 374) and genomic (*n* = 460) sequencing data. We then validated the results on an external cohort of 135 NDMM patients with CTCs enumerated by next‐generation flow cytometry. Higher CTC levels were significantly associated with high‐risk clinical features (e.g., ISS or IMS/IMWG 2024). Furthermore, genomic analyses revealed that high CTC counts were associated with complex genomic features such as chromothripsis, APOBEC mutagenesis, and loss of key tumor suppressors, typically linked to high‐risk disease. Transcriptomic analyses revealed that elevated CTCs were enriched in cell cycle and proliferation (PR) genes while presenting a reduced association with immune response. Importantly, CTCs also emerged as a surrogate for PR transcriptomic signatures and demonstrated prognostic superiority, potentially simplifying application in the clinical setting. Elevated CTC levels reflect aggressive biological features of multiple myeloma and outperform prognostic markers such as PR signatures. Integrating CTC data into genomic and transcriptomic classifiers could enhance risk stratification and provide a streamlined and powerful tool for clinical decision‐making in NDMM.

## Graphical abstract

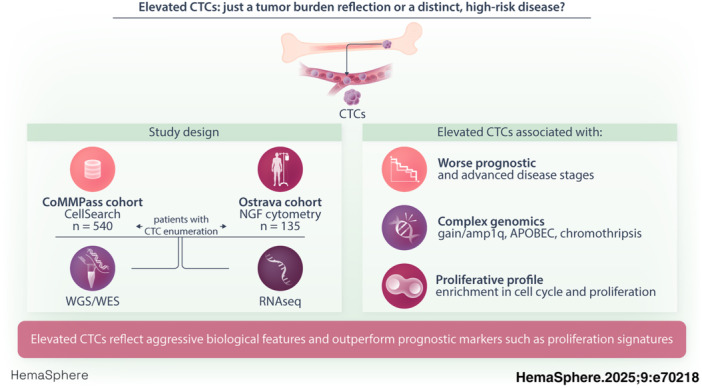

## INTRODUCTION

Multiple myeloma (MM) is a genetically heterogeneous hematological malignancy typically characterized by the proliferation (PR) of clonal plasma cells (PCs) within the bone marrow (BM). In a majority of patients, however, clonal PCs are able to egress from BM to peripheral blood (PB) as circulating tumor cells (CTCs), suggesting an association with disease dissemination or aggressiveness.^1^, ^2^ Consequently, CTC quantification is emerging as a relevant prognostic factor in MM, showing clinical significance across all stages of disease evolution from smoldering to active MM and plasma cell leukemia (PCL).^3^, ^4^, ^5^, ^6^, ^7^

While previously published data suggested a correlation between CTC levels and BM PC infiltration (i.e., tumor burden), elevated CTCs can also be observed in patients with low BM involvement and vice versa.^3^, ^4^ This observation indicates that CTCs may reflect an intrinsic disease biology driving BM PC egression rather than being solely a reflection of tumor burden. Indeed, besides classical disease burden markers, high CTC levels have been independently associated with high‐risk chromosomal aberrations evaluated by fluorescence in situ hybridization (FISH), such as amp1q/del1p, del17p, or t(4;14).^4^

As MM diagnostic profiling and risk definition are evolving from cytogenetics to genomics and transcriptomics,^8^, ^9^, ^10^ it is important to determine how CTCs relate to key determinants of high‐risk disease and whether they possess independent prognostic value to improve MM risk stratification. A few studies based on patient‐paired genomic and transcriptomic profiling of BM PCs and CTCs demonstrated a high concordance in somatic mutations and copy number variations, suggesting a strong clonal relationship irrespective of anatomical location.^11^, ^12^, ^13^, ^14^ Nevertheless, there is only limited knowledge about specific genomic and transcriptomic features associated with PC egression into circulation. A more comprehensive understanding of the mechanisms responsible for the extent of CTC burden is essential for improved prognostication and the development of targeted strategies against disease dissemination.

To investigate these relevant aspects, we comprehensively integrated transcriptomic and genomic information of BM samples from 540 newly diagnosed MM (NDMM) patients with available CTC enumeration in the CoMMpass dataset.^10^ Additionally, we validated our findings on an independent dataset of 135 NDMM patients with CTCs assessed by next‐generation flow (NGF) cytometry. Here, we confirmed the clinical impact of CTC levels and revealed, through two different approaches, that they are associated with distinct high‐risk genomic and transcriptomic profiles. Importantly, CTCs also emerged as a surrogate and superior prognostic marker for the RNA sequencing (RNAseq)‐based PR signature,^8^, ^9^, ^10^ potentially simplifying the application of this important prognostic marker in the clinical setting. Altogether, these data support CTC assessment as a powerful and readily applicable approach for identifying high‐risk NDMM.

## METHODS

**Figure 1:**
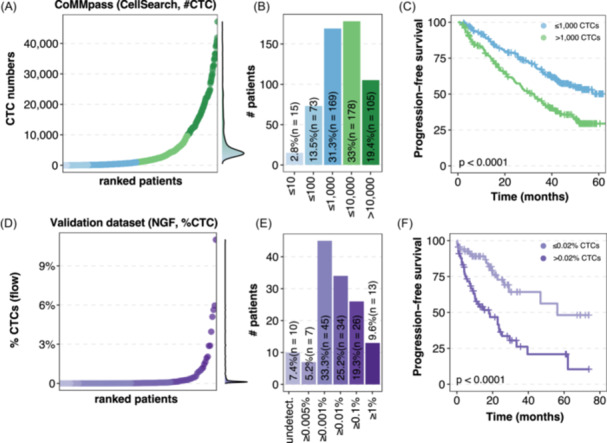
**Circulating tumor cell (CTC) levels in newly diagnosed multiple myeloma (NDMM) patients identify different prognostic groups. (A, D)** NDMM patients ranked by CTC levels. CTCs were enumerated through CellSearch in CoMMpass (**A**, *n* = 540) or next‐generation flow (NGF) cytometry in the validation dataset (**D**, *n* = 135). Increasing colors represent CTC categorization by logarithmically based increments. Side densities show the unordered CTC distribution. **(B, E)** Patient numbers according to incrementing logarithmic CTC levels both for discovery **(B)** and validation **(E)** datasets. Undetect., undetectable CTCs (below limit of detection). **(C, F)** Kaplan–Meier curves for progression‐free survival (PFS) for CoMMpass **(C)** and validation dataset **(F)**. Patients are stratified according to median CTC values (i.e., 1000 CTCs and 0.02% CTCs for CoMMpass and validation datasets).

This study comprises two independent datasets of NDMM patients with available CTC assessments (Supporting Information S1: Figure 1 and Supporting Information S3: Table 1). A total of 540 patients included in the CoMMpass dataset (NCT01454297) with CTCs enumerated at baseline through the CellSearch System constitutes the discovery cohort.^15^ Briefly, this protocol magnetically enriches PB cells based on CD138 expression, followed by additional staining with CD45, CD19, CD38, and 4',6‐diamidino‐2‐phenylindole. Cells positive for CD38 and negative for CD45 and CD19 are considered CTCs. Additionally, RNAseq and whole‐genome/exome sequencing (WGS/WES) information were available from CD138‐enriched PCs in 374 and 460 patients, respectively.

The validation dataset from the University Hospital Ostrava (Czech Republic) comprised 135 NDMM patients; CTCs were evaluated by NGF according to the Euroflow protocol.^16^ In addition, RNAseq and WGS were generated from 60 and 12 patients, respectively. Samples were manually selected to fairly represent each CTC‐based logarithmic group (i.e., undetectable CTCs, ≥0.0005%, ≥0.001%, ≥0.01%, ≥0.1%, ≥1%, and ≥10% CTCs), and BM tumor cells were fluorescence‐activated cell sorted according to their patient‐specific aberrant phenotype.^17^

Spearman's, Pearson's, Kruskal–Wallis', and Dunn's statistical tests were used to study correlations and enrichment between CTC levels and clinical, genomic, and transcriptomic features. Analyses for progression‐free (PFS) or overall survival (OS) were based on log‐rank tests and Kaplan–Meier curves. The full description of the Materials and Methods is available in the Supporting Information S2: Data Supplement.

### Data sharing statement

All the data presented in this article are available upon reasonable request to the corresponding author. CoMMpass data are publicly available through the Multiple Myeloma Research Foundation Research Portal. Sequencing raw data from the validation dataset are available in EGA (WGS: EGAD50000001724; RNA: EGAD50000001725).

## RESULTS

### Elevated CTC levels are associated with high‐risk clinical features

**Figure 2:**
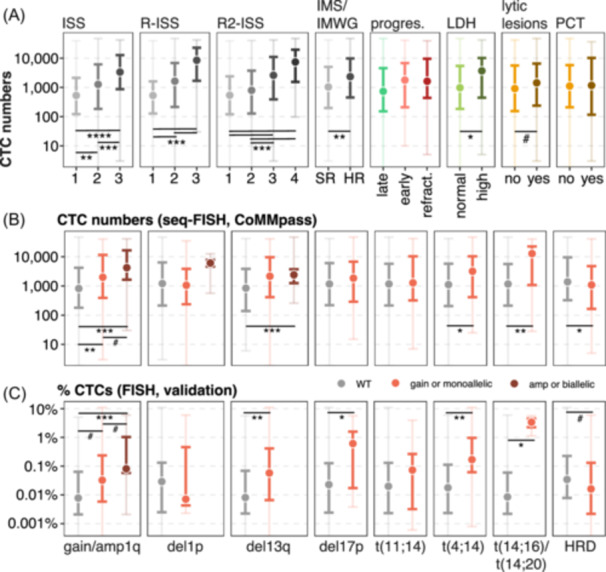
**Elevated circulating tumor cell (CTC) numbers were associated with high‐risk clinical features. (A)** CTC distribution according to the International Staging System (ISS), the Revised‐ISS (R‐ISS), the R2‐ISS, the new International Myeloma Society/International Myeloma Working Group (IMS/IMWG) risk definition, late versus early progressors (i.e., ≤18 months), lactate dehydrogenase (LDH) levels, and the presence of lytic lesions or para‐skeletal/extramedullary plasmacytomas (PCT). CTC numbers were log‐10 transformed for visualization purposes. See Supporting Information S4: Table 2 for detailed statistics. **(B, C)** CTC distribution according to cytogenetic aberrations assessed by seq‐FISH (CoMMpass, **B**) or classical fluorescence in situ hybridization (FISH, validation dataset, **C**). gain (3 copies); amp, amplification (>3 copies); del, deletion; HR, high risk; HRD, hyperdiploidy; refract., refractory; SR, standard risk; t, translocation. ****P ≤ 0.0001; ***P ≤ 0.001; **P ≤ 0.01; *P ≤ 0.05; ^#^P ≤ 0.1.

**Figure 3:**
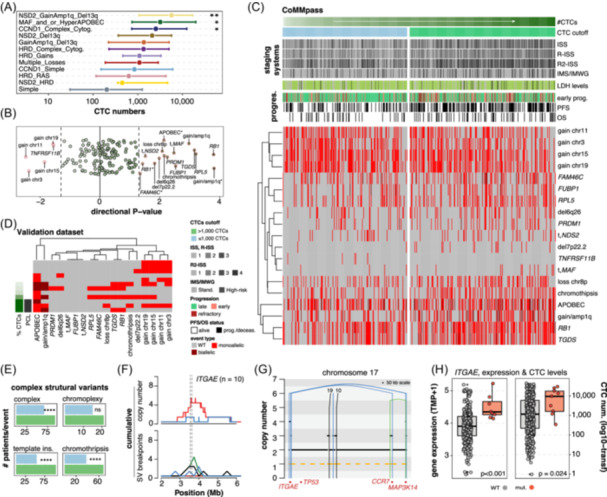
**Patients with elevated circulating tumor cell (CTC) levels presented higher frequencies of complex genomic events. (A)** CTC distribution according to the genomic classification from the IRMMa model.^21^ CTC numbers were log‐10 transformed for visualization purposes. All comparisons are against the “Simple” group as reference. **(B)** Directional P‐value depiction of IRMMa genomic features according to their association with CTC levels. The darker the color, the higher the number of CTCs. Only significant features (P ≤ 0.05, in red) were annotated (see Supporting Information S4: Table 7 for the complete list). Features with asterisks denote amplification (i.e., ≥4 copies of 1q), hyper‐APOBEC, or biallelic inactivation (for punctual genes). P‐value adjustment kept the same gene enrichment pattern with the exception of *FAM46C* (adj. P = 0.13; Supporting Information S4: Table 4). **(C)** Unsupervised clustering ordered by CTC numbers and based on the previous 19 significant features for CoMMpass. Gray, light, and dark red colors indicate no event (i.e., wild‐type, WT), monoallelic, or biallelic events, respectively, except in the case of gain/amp1q and APOBEC, where dark red denotes amplification and hyper‐APOBEC. The green gradient corresponds to CTC counts or % for CellSearch and flow cytometry, respectively. Additional risk factors such as International Staging System (ISS), lactate dehydrogenase (LDH) levels, progression‐free survival (PFS), or overall survival (OS) status, among others, were added to the annotation. **(D)** Unsupervised clustering based on the previous 19 significant features for the validation dataset. **(E)** Number of cases with complex events (i.e., chromoplexy, chromothripsis, complex, and template insertions) when categorizing by more (green) or less (blue) than 1000 CTCs. **(F)** *ITGAE* was the only significant gene affected by structural gains in hotspot analyses, as the punctual example depicts **(G)**. **(H)** Expression levels and CTC counts according to *ITGAE* mutation status. Notably, only one case co‐occurred with a *TP53* aberration. IMS, International Myeloma Society; IMWG, International Myeloma Working Group; PCL, plasma cell leukemia; SV, structural variants. ****P ≤ 0.0001; ***P ≤ 0.001; **P ≤ 0.01; *P ≤ 0.05﻿﻿.

**Figure 4:**
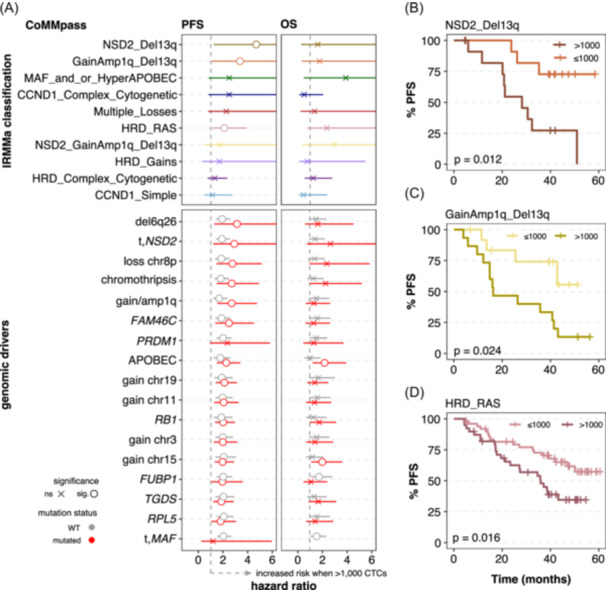
**Circulating tumor cells (CTCs) added an important prognostic value to current genomic‐based stratification. (A)** Subgroup analysis of progression‐free survival (PFS) and overall survival (OS) for IRMMa's genomic groups (top part) and significant genomic features (bottom part). The aberration status for each genomic group or driver (wild‐type, WT, in orange, or mutated, in gray) was stratified according to the CTC level (≤/>1000 CTCs). “x” depicts nonsignificant (unadjusted P > 0.05). Genomic groups *Simple* and *NSD2_HRD*, as well as features *TNFRSF11B* and del7p, were removed due to very few cases with survival data for PFS or OS. **(B–D)** Kaplan–Meier curves for PFS for significant IRMMa's genomic groups. Patients were stratified according to the presence of high or low CTC levels (i.e., 1000 CTCs).

For 540 NDMM patients from the CoMMpass dataset, the median number of CTCs was 1132, and values ranged from zero to 47,146 CTCs following a marked exponential distribution (Figure 1A). More specifically, two patients (0.4%) had no CTCs, and 2.4%, 13.5%, 31.3%, 33%, and 19.4% of patients presented ≤10, ≤100, ≤1000, ≤10,000, and >10,000 CTCs, respectively (Figure 1B). The presence of CTCs above the median (i.e., 1000 CTCs) showed significant prognostic value in terms of PFS (log‐rank test, P < 0.001; Figure 1C). The flow‐based validation dataset displayed a more skewed distribution of CTCs, likely due to the increased resolution of NGF as compared to CellSearch (Figure 1D). However, the group distribution according to incrementing CTC levels was similar (Figure 1E). Again, the median (i.e., 0.02%) was able to identify two very different groups according to PFS (log‐rank test, P < 0.001; Figure 1F). Although not significant, these CTC‐based cutoffs displayed a separating trend also for OS (Supporting Information S1: Figure 2). Transformed CTC counts from the NGF percentage showed a distribution pattern very similar to that of the CoMMpass dataset (Supporting Information S1: Figure 3). Interestingly, and corroborating previous NGF reports,^18^, ^19^ both datasets showed a group of patients with low CTC levels (≤10 CTCs, *n* = 15, for CoMMpass; or <0.001% CTCs, *n* = 17, for the validation dataset) characterized by favorable outcomes without any documented death (log‐rank test, P < 0.05; Supporting Information S1: Figure 4).

**Figure 5:**
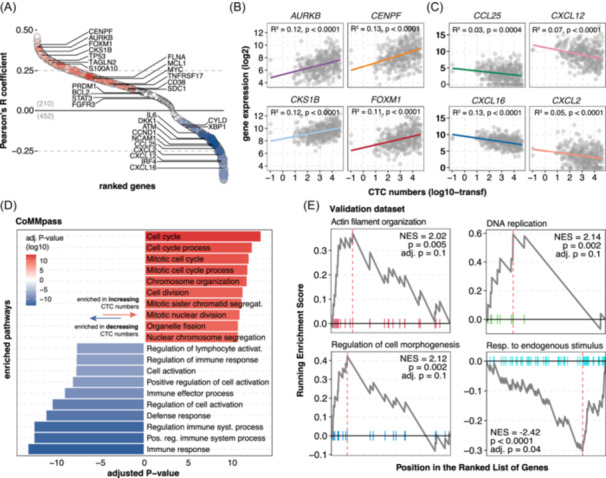
**Elevated circulating tumor cell (CTC) numbers showed a transcriptomic enrichment in cell cycle‐related functions and a reduction in immune system activation. (A)** Ranked genes according to their Pearson's *R* coefficient. Red‐blue gradient depicts the log‐2 fold‐change (log2FC) from positive to negative values; dot sizes are linked to the adjusted P‐values. See Supporting Information S4: Table 4 for the complete gene list. **(B, C)** Examples of positively **(B)** and negatively **(C)** correlated genes according to CTC numbers (log‐10 transformed). **(D)** Top 10 enriched functions in a gene‐set enrichment analysis (GSEA) separately performed for positively and negatively correlated genes. Color and bar sizes indicate the adjusted P‐values (log‐10 transformed). **(E)** Punctual GSEA validation in the external dataset. NES, normalized enrichment score.

**Figure 6:**
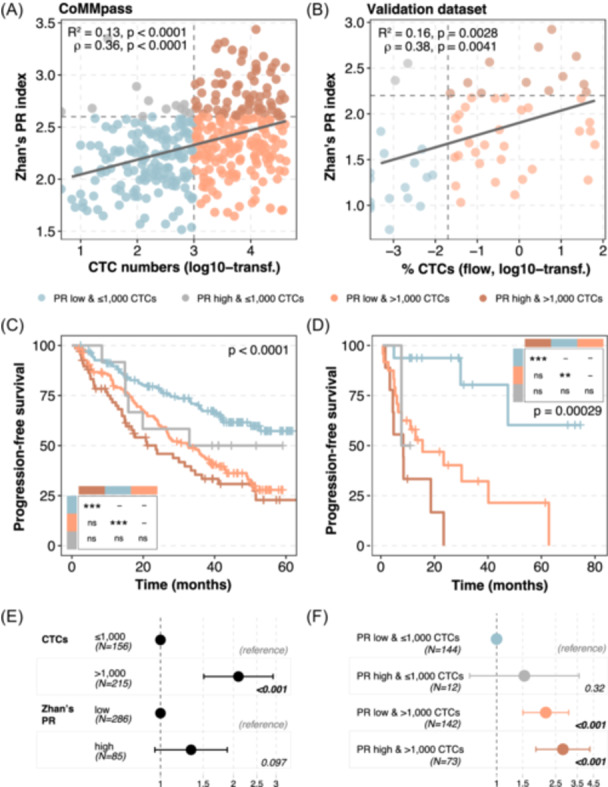
**Circulating tumor cell (CTC) levels reflected proliferation (PR) signatures and had a worse combined prognostic value. (A, B)** Correlations between Zhan's PR signature and CTC levels assessed by CellSearch **(A)** or next‐generation flow (NGF) cytometry **(B)**. Cutoffs are the median of CTC levels (1000 CTCs or 0.02% for the CoMMpass and the validation datasets, respectively) and the third quartile of the PR signature. **(C, D)** Kaplan–Meier curves for progression‐free survival (PFS) stratifying patients according to PR and CTC levels for CoMMpass **(C)** and validation **(D)** datasets. Colors follow the patient distribution in **(A, B)**. ****P ≤ 0.0001; ***P ≤ 0.001; **P ≤ 0.01; ns, nonsignificant (i.e., P > 0.05). **(E, F)** Multivariate analysis on PFS for Zhan's PR and CTCs cutoffs (i.e., 2.6 and 1000 CTCs, respectively; **E**) and their combination in the four subsets of patients **(F)**.

Elevated CTCs were strongly associated with high‐risk MM defined according to the International Staging System (ISS), the Revised ISS (R‐ISS), and the R2‐ISS (Dunn's test, P ≤ 0.001), as well as with the new risk definition of the International Myeloma Society and International Myeloma Working Group (IMS/IMWG; Kruskal's test, P = 0.003).^20^ The incorporation of CTC levels into each stratifying system further improved their prognostic and discriminating power (i.e., concordance c‐index; Supporting Information S1: Figure 5). Functional high‐risk MM patients (i.e., progression within 18 months) also presented a slight, but not significant, increment in their CTC counts (Dunn's test, P = 0.12; Figure 2A and Supporting Information S4: Table 2). BM tumor cell infiltration and CTC numbers showed a significant correlation (Spearman's *ρ* = 0.4, P < 0.001; Supporting Information S1: Figure 6A). A similar pattern was observed with ß2‐microglobulin (Spearman's *ρ* = 0.33, P < 0.001) but not for the monoclonal component (Spearman's *ρ* = 0.09, P = 0.086; Supporting Information S1: Figure 6B,C). Moreover, while elevated LDH levels were associated with high CTCs (Kruskal's test, P = 0.02), the presence of lytic lesions or plasmacytomas did not significantly correlate with CTC counts (Figure 2A and Supporting Information S4: Table 2). Among the common cytogenetic abnormalities tested by either seq‐FISH (CoMMpass) or FISH (validation), gain/amp1q and del13q presented high CTCs in both the CoMMpass and validation datasets, respectively (Dunn's test, P < 0.001 and P ≤ 0.005). Translocations involving *MAF/MAFB* (i.e., t(14;16) or t(14;20)) or *NSD2* (i.e., t(4;14)) were also associated with high CTC counts (Kruskal's test, P ≤ 0.03 in all cases). Nevertheless, del17p was only significant in the validation dataset but not in CoMMpass, and del1p was not associated with CTC numbers in either of the two datasets (Figure 2B,C and Supporting Information S4: Table 3).

### Patients with high CTC levels are enriched for high‐risk genomic features

Of the 540 patients with CTC data in the CoMMpass dataset, 460 patients had available genomic data. We examined the influence of genomic features on CTC levels in the context of recently defined MM molecular subgroups derived from an analysis of almost 2000 patients (IRMMa).^21^ Those subgroups characterized by high genomic complexity (i.e., combinations of complex structural variation, loss of multiple tumor suppressors, and high APOBEC contribution) including *NSD2_GainAmp1q_Del13q* (Dunn's test, adj. P = 0.007), *MAF_and/or_HyperAPOBEC* (adj. P = 0.013), and *CCND1_Complex_Cytogenetics* (adj. P = 0.02), displayed elevated CTC numbers (Figure 3A and Supporting Information S4: Table 4). Upon individually analyzing 113 genomic features included in the recent genomic classification, 19 were revealed to be correlated with CTC counts (i.e., P ≤ 0.05; Figure 3B,C and Supporting Information S4: Table 4). Overall, genomic features known to be of prognostic importance were associated with high CTC egression potential including gain/amp1q, *MAF/MAFB* and *NSD2* translocations, APOBEC mutagenesis, deletions of 6q or 8p, chromothripsis (as defined by copy number signature^22^), and mutations in several tumor suppressor genes (*RB1*, *RPL5, FUBP1*, *PRDM1*, and *FAM46C*). Cases with low CTC numbers typically presented, by contrast, a higher frequency of gains in chromosomes 3, 11, 15, and 19 (i.e., hyperdiploid‐like; Supporting Information S1: Figure 7), and mutations in *TNFRSF11B*.

Despite the limited number of cases (*n* = 12), a similar pattern was observed in the validation dataset, where the samples were selected to represent patients with extremely high or low/undetectable levels of CTCs. Again, patients with elevated CTCs presented high APOBEC mutagenesis and gain/amp1q. Notably, the validation set included three cases of PCL, as defined by ≥20% CTCs, and these cases emphasized these associations with enrichment for hyper‐APOBEC and gain/amp1q (Figure 3E).

The recently proposed genomic classification for NDMM was based on WES and targeted sequencing data and, aside from *IGH* translocations, it did not account for other structural variants (SVs) and complex SV events.^21^ Thanks to the availability of WGS, we were able to investigate the relationship between CTC levels and these critical features. Specifically, chromothripsis and complex not‐otherwise‐specified SVs—but not chromoplexy—were enriched in cases with high CTC levels (chi‐squared test, P < 0.0001; Figure 3F). Aside from *IGH* translocations, only one known SV hotspot out of 68 (i.e., *nonClustered_chr17_3.8Mb* ^23^) emerged with a higher‐than‐expected amplification density in cases with elevated CTC counts (*n* = 10; Wilcoxon's test, adj. P = 0.023; Figure 3G,H). SVs involving this hotspot resulted in the amplification and overexpression of *ITGAE* and were related to increased CTCs (Wilcoxon's test, P ≤ 0.024; Figure 3H).^24^, ^25^, ^26^, ^27^ Interestingly, this gene is highly expressed in hairy cell leukemia and some T‐cell lymphomas, as well as in tumor‐infiltrating T‐regulatory cells, and has an important role in interacting with Type I conventional dendritic cells, supporting MM progression.^24^, ^25^, ^26^, ^27^ Altogether, these observations reveal a marked association between complex and aggressive BM genomic features and high circulating tumor burden.

### Improved resolution of genomic‐based risk stratification with incorporation of CTC levels

Given the prognostic potential of CTCs and the relevance of genomic features in patient risk stratification, we next evaluated their combined impact on clinical outcomes. The presence of more than 1000 CTCs significantly improved the prognostic value for PFS of IRMMa's genomic groups *NSD2_Del13q* (hazard ratio [HR] = 4.7, P = 0.012), *GainAmp1q_Del13q* (HR = 3.4, P = 0.024), and *HRD_RAS* (HR = 2.1, P = 0.016), effectively resolving those genomic subgroups by high‐risk biological behavior (Figure 4A–D and Supporting Information S4: Table 5).

The presence of more than 1000 CTCs significantly increased the risk of progression globally, regardless of the genomic event status (wild‐type, mutated, or multi‐allelic). After excluding two features with five or fewer cases with survival information (2/19), the combination of CTCs with each of these events revealed a significantly cumulative prognostic effect in 11 of the 17 genomic features previously linked to increasing CTC numbers (Figure 4A, bottom; and Supporting Information S1: Figure 8). Poor prognosis was particularly associated with patients presenting *NSD2*‐related translocations (HR = 2.92), gain/amp1q (HR = 2.71), chromothripsis (HR = 2.71), *FAM46C* (HR = 2.50), and APOBEC mutagenesis (HR = 2.27). In contrast, hyperdiploid‐related gains offered only modest prognostic benefit, mainly driven by CTC levels. Notably, among all features, only the combination of CTCs and APOBEC mutagenesis had a consistent and significant impact on OS (HR = 2.17). Overall, these results suggest that genomic‐based patient stratification may benefit from incorporating CTC enumeration.

### CTCs are correlated with specific transcriptomic signatures

Due to the particular association of CTCs with BM tumor burden (Supporting Information S1: Figure 6A) and their distribution in the CoMMpass dataset, we investigated the transcriptomic features linked with CTC levels using a linear model adjusted for BM PC infiltration.^4^, ^5^, ^9^ A total of 662 genes displayed a significant association with CTC numbers (adj. P ≤ 0.01; Figure 5A and Supporting Information S4: Table 6). Of these, 210 genes such as *AURKB*, *FLNA, TAGLN2, S100A10, ENO1*, *CENPF*, *CKS1B*, or *FOXM1*, positively correlated with CTC numbers (Figure 5A,B and Supporting Information S1: Figure 9A) and showed enrichment in functions related to the cell cycle and PR (e.g., mitotic‐related processes, cell division, or chromatid segregation; adj. P < 0.0001; Figure 5D and Supporting Information S4: Table 8). The validation dataset confirmed these results and expanded them with additional pathways including regulation of cell adhesion or ribosome biogenesis (adj. P = 0.1; Figure 5E and Supporting Information S4: Tables 7 and 9). In line with the cytogenetic and genomic associations with gain/amp1q, these positively correlated genes tended to show a distribution clustering in that chromosome region (Supporting Information S1: Figure 10).

Among the other 452 genes with inversely correlated expression, the top enriched pathways involved immune system regulation (e.g., immune response processes, or cell activation; adj. P < 0.0001; Figure 5D and Supporting Information S4: Tables 6–9). Interestingly, among these genes, there were several cytokines, such as *CCL25*, *CXCL12*, *CXCL16*, or *CXCL2* (Figure 5C and Supporting Information S1: Figure 9B), further implicating migration‐related functions as important for egression (e.g., response to cytokines, locomotion, or regulation of cell–cell adhesion). In general, high CTC levels showed a positive correlation with genes involved in PR and cell cycling, while they were negatively enriched in functions associated with immune system activation and cell adhesion.

### Elevated CTC levels reflect the tumor PR gene expression signature

PR signatures have long been recognized as a powerful marker of high‑risk disease, though they remain challenging to implement in routine clinical practice.^8^, ^9^, ^10^ To address this, we evaluated whether CTC levels could serve as a surrogate indicator for PR signatures. We tested the relationship of CTCs with the PR signatures in MM from Zhan et al.^8^ and Skerget et al.^10^ and observed a significant association for both signatures (Spearman's *ρ* = 0.36 and *R* ^2^ = 0.13, Figure 6A; Spearman's *ρ* = 0.26 and *R* ^2^ = 0.06, Supporting Information S1: Figure 11A; P < 0.001). The validation dataset corroborated these results (Spearman's *ρ* = 0.38 and *R* ^2^ = 0.16, Figure 6B; Spearman's *ρ* = 0.33 and *R* ^2^ = 0.1, Supporting Information S1: Figure 11B; P ≤ 0.02). The same pattern was observed for the PCL‐like transcriptomic classifier recently developed (Spearman's *ρ* = 0.38 and *ρ* = 0.57; *R* ^2^ = 0.13 and *R* ^2^ = 0.3; for discovery and validation datasets, respectively, P < 0.001; Supporting Information S1: Figure 11C,D).^9^

Importantly, the combination of CTC counts with Zhan's PR demonstrates that CTC quantification offers a more practical and potentially superior prognostic tool—one that could replace PR signatures in clinical practice (log‐rank test, P < 0.0001; Figure 6C). Specifically, while patients with low CTC counts and low PR signature had the most favorable outcomes (PFS not reached, *n* = 144), and those with elevated CTCs and high PR index experienced significantly shorter PFS (24 months, *n* = 73), a third subgroup—patients with high CTC counts but low PR signature—showed similarly poor outcomes with a median PFS of 33 months (*n* = 142). Only 12 patients had elevated PR index and low CTCs, with a negative predictive value of 34%. Upon examining the OS, only the combination of high CTCs and PR values retained significant prognostic value (P = 0.0005; Supporting Information S1: Figure 12). The validation dataset displayed similar survival patterns for PFS (log‐rank test, P = 0.0003; Figure 6D). In all cases, the presence of elevated CTC values—alone and in combination—retained the higher prognostic effect and substantially improved PR value (log‐rank test, P < 0.001; Figure 6E,F). Overall, these findings demonstrate that elevated CTC levels reflect highly proliferative tumor behavior and could reliably substitute PR signatures in clinical practice.

## DISCUSSION

CTCs are emerging as a highly important prognostic feature for risk stratification in MM.^4^, ^5^, ^7^ We confirm here that high CTC levels assessed by NGF and count‐based approaches—the largest dataset to date—were able to identify different prognostic groups. Our analysis showed that, while NGF offers higher resolution through the detection of CD45^+^ MM cases and clonality assessment compared to the CellSearch System, the prognostic value of CTCs by both approaches is useful for predicting PFS. These data strongly support the hypothesis of CTCs as a marker of tumor aggressiveness, and not just tumor burden. Nevertheless, it has remained unclear whether CTCs are a simple indication of underlying disease biology or if they provide prognostic utility independent of that offered by comprehensive genomic and transcriptomic profiling. To investigate these key questions, we examined two independent datasets of NDMM patients and showed that elevated CTC levels presented enrichment for distinct genomic features. Specifically, patients with high CTC levels presented a more aggressive genomic profile characterized by combinations of high‐risk genomic features including gain/amp1q, *NSD2‐* or *MAF/MAFB*‐related translocations, APOBEC mutagenesis, chromothripsis, and complex SVs. Importantly, while t(4;14) or del13q are not necessarily associated with high genomic complexity, patients with hyper‐APOBEC activity are known to have a highly complex and aggressive profile.^28^ The combination of these genomic features with CTC levels provided enhanced resolution to identify those patients with more biologically aggressive disease.

The strongest association was observed at the transcriptomic level, where elevated CTCs correlated with increased PR and cell cycle‐related functions, alongside a reduction in the expression of adhesion molecules and a potential immune system activation. A recent single‐cell RNAseq study on paired CTCs and BM did not show major differences, suggesting that the key transcriptional networks are preserved once the PCs egress the medullary milieu.^29^ However, further studies are needed to support these biological results. Cell cycle may explain the elevated CTC counts observed, and its activity has long been considered a strong prognostic marker in NDMM, with various PR gene expression signatures developed over the last 20 years.^8^, ^9^, ^10^, ^30^ Although these signatures accurately predict high‐risk disease, their clinical application has been limited by the logistical challenges associated with generating gene expression and RNAseq data. Our data demonstrated an association between CTCs and these PR signatures. More importantly, by combining them, we were able to define a group of patients with a low PR signature and high CTC levels with reduced survival, suggesting that CTCs recapitulate the specificity of PR signatures while enhancing their sensitivity. CTC enumeration emerges here as a minimally invasive, economical, logistically favorable, and accurate prognostic feature for identifying high‐risk disease.

With the increasing relevance of genomic profiling for individualized patient risk assessment, CTC assessment could be a minimally invasive and reproducible approach for adding further phenotypic resolution. Besides showing a clear association with high‐risk clinical scenarios, we also demonstrated a significant association of CTC levels with high levels of cellular PR (or cell cycling) and a high‐risk and more complex genomic profile.^21^, ^22^, ^23^ The proof‐of‐concept combining CTCs with some of the IRMMa key features improved their prognostic potential, suggesting that the addition of CTC enumeration may further polish genomic‐based risk stratification of MM patients. In this refinement process, the incorporation of the treatment will be also essential for treatment monitoring (Supporting Information S1: Figure 13). Nevertheless, due to the absence of patients in CoMMpass who received anti‐CD38 monoclonal antibody‐based regimens, future studies will be necessary to evaluate the prognostic utility of CTCs in the context of immunotherapy.

Overall, our study confirms that CTC levels are associated with multiple risk factors and represent a powerful prognostic marker for clinical outcomes in NDMM. They retain their prognostic significance independently of the latest high‑risk genomic and clinical groupings and could serve as a surrogate for markers of high‑risk disease, tumor burden, and PR.

## AUTHOR CONTRIBUTIONS

**Juan‐Jose Garces**: Conceptualization; methodology; data curation; investigation; formal analysis; software; visualization; writing—original draft; writing—review and editing. **Benjamin Diamond**: Conceptualization; methodology; formal analysis; funding acquisition; visualization; writing—review and editing; writing—original draft; investigation. **Tereza Sevcikova**: Conceptualization; methodology; investigation; formal analysis; validation; writing—review and editing. **Serafim Nenarokov**: Methodology; formal analysis; writing—review and editing; software. **Daniel Bilek**: Methodology; software; formal analysis; writing—review and editing. **Eva Radova**: Resources; writing—review and editing. **Ondrej Venglar**: Resources; writing—review and editing. **Veronika Kapustova**: Resources; writing—review and editing. **Ross Firestone**: Resources; writing—review and editing. **Kylee Maclachlan**: Resources; writing—review and editing. **Anish Simhal**: Resources; writing—review and editing. **Lucie Broskevicova**: Resources; writing—review and editing. **Jan Vrana**: Resources; writing—review and editing. **Ludmila Muronova**: Resources; writing—review and editing. **Tereza Popkova**: Resources; writing—review and editing. **Jana Mihalyova**: Resources; writing—review and editing. **Hana Plonkova**: Resources; writing—review and editing. **Michael Durante**: Resources; writing—review and editing. **Bachisio Ziccheddu**: Resources; writing—review and editing. **Michal Simicek**: Resources; writing—review and editing. **Hearn Jay Cho**: Resources; writing—review and editing. **George Mulligan**: Resources; writing—review and editing. **Jonathan Keats**: Resources; writing—review and editing. **David Zihala**: Supervision; resources; writing—review and editing; formal analysis; methodology. **Ola Landgren**: Supervision; resources; writing—review and editing. **Roman Hajek**: Supervision; resources; writing—review and editing. **Saad Usmani**: Supervision; writing—review and editing; resources. **Francesco Maura**: Conceptualization; methodology; investigation; formal analysis; supervision; funding acquisition; visualization; writing—review and editing. **Tomas Jelinek**: Conceptualization; methodology; formal analysis; funding acquisition; supervision; writing—review and editing.

## CONFLICT OF INTEREST STATEMENT

B.D. reports consulting from Janssen and Sanofi.

S.U. reports research funding from AbbVie, Amgen, BMS/Celgene, GSK, Gilead, Janssen, Merck, Pharmacyclics, Sanofi, Seattle Genetics, SkylineDX, and Takeda; consulting for AbbVie, Amgen, BMS/Celgene, Genentech, GSK, Janssen, Oncopeptides, Pfizer, Sanofi, Seattle Genetics, SecuraBio, SkylineDX, Takeda, and TeneoBio.

F.M. reports consulting for Medidata and Sanofi.

The rest of the authors declare no conflicts of interest.

## FUNDING

This work was supported by the European Union project LERCO (No. CZ.10.03.01/00/22_003/0000003), the Czech Health Research Council (NU23‐03‐00374), and the Ministry of Education, Youth and Sports, OPJAK (CZ.02.01.01/00/22_008/0004644); institutional support (MH CZ‐DRO‐FNOs/2022, MH CZ‐DRO‐FNOs/2023) from the Ministry of Health of the Czech Republic; the students' grant system of the University of Ostrava (SGS03/PRF/2025, SGS20/LF/2025); and the Ministry of Education, Youth and Sports of the Czech Republic through e‐INFRA CZ (ID: 90254). It was also supported by the Sylvester Comprehensive Cancer Center National Cancer Institute (NCI) Core Grant (P30 CA 240139) and the Memorial Sloan Kettering Cancer Center NCI Core Grant (P30 CA 008748). B.D. is a K12 Scholar supported by the NCI‐NIH under Award Number K12CA226330.

K.M. has received funding from the Multiple Myeloma Research Foundation, the American Society of Hematology, and the International Myeloma Society. T.S. and M.S. are supported by the Technology Agency of the Czech Republic (TQ03000338). F.M. is supported by the Department of Defense (DoD) and the NIH‐NCI (R37CA289752‐01A1). T.J. is supported by the EHA Physician Scientist Research Grant (RG‐202409‐06772).

## Supporting information

Supporting Information.

Supporting Information.

Supporting Information.

Supporting Information.

## Data Availability

The data that support the findings of this study are openly available in EGA at https://ega-archive.org/.
