# Supplementary material for: Elevated circulating tumor cells reflect high proliferation and genomic complexity in multiple myeloma
Source: Hemasphere. 2025 Sep 23;9(9):e70218. doi: 10.1002/hem3.70218 (PMC12455875; doi:10.1002/hem3.70218)
Supplement: Supplementary file 1 — Supporting Information. [file HEM3-9-e70218-s002.docx]

**Supplemental Figures for:**

**Elevated circulating tumor cells reflect high proliferation and genomic complexity in multiple myeloma**

**Juan-Jose Garces, Benjamin Diamond, Tereza Sevcikova, Serafim Nenarokov, Daniel Bilek, Eva Radova, Ondrej Venglar, Veronika Kapustova, Ross Firestone, Kylee Maclachlan,** Anish Simhal**, Lucie Broskevicova, Jan Vrana, Ludmila Muronova, Tereza Popkova, Jana Mihalyova, Hana Plonkova, Michael Durante, Bachisio Ziccheddu, Michal Simicek, Hearn Jay Cho, George Mulligan, Jonathan Keats, David Zihala, Ola Landgren, Roman Hajek, Saad Usmani, Francesco Maura, Tomas Jelinek**

| **INDEX** |  |
| --- | --- |
| **Figure 1. CONSORT diagram.** | **2** |
| **Figure 2. KM curves for OS for CoMMpass and validation datasets, median CTC cutoffs.** | **3** |
| **Figure 3. Absolute CTC counts distributions.** | **4** |
| **Figure 4. KM curves for PFS and OS for CoMMpass and validation dataset, logarithmic increments.** | **5** |
| **Figure 5. PFS KM curves from combining CTCs and other stratifying systems.** | **6** |
| **Figure 6. CTCs vs BM tumor infiltration, B2M and M-protein.** | **7** |
| **Figure 7. CNV profile for CTCs below and above 1,000 counts.** | **8** |
| **Figure 8. KM curves for PFS for prognostically significant IRMMa’s features.** | **9** |
| **Figure 9. Positively and negatively CTC correlated genes in validation dataset.** | **10** |
| **Figure 10. Chromosome regions enrichment for DEGs.** | **11** |
| **Figure 11. CTCs vs other proliferation signatures.** | **12** |
| **Figure 12. KM curves for OS according to PR and CTC levels.** | **13** |
| **Figure 13. KM curves for PFS stratified by VRD or other treatments.** | **14** |

**Supplemental Figure 1.** CONSORT diagram.


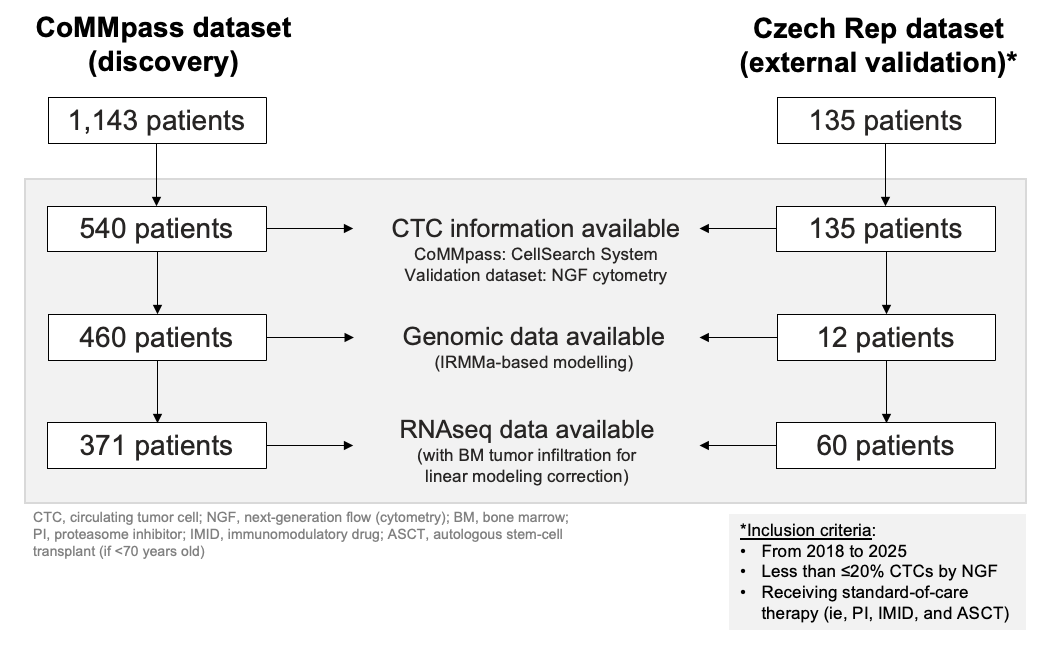


**Supplemental Figure 2.** Kaplan-Meier curves for OS for CoMMpass (**A**) and validation dataset (**B**). Patients are stratified according to median CTC values (i.e., 1,000 CTCs and 0.02% CTCs for CoMMpass and validation datasets, respectively).

**
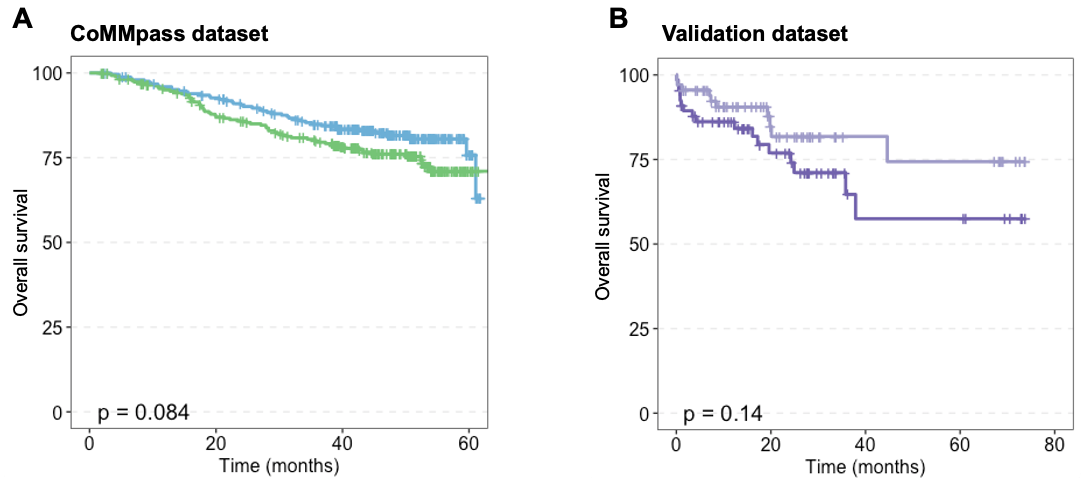
**

**Supplemental Figure 3.** Flow-based percentages converted to counts/μl and overlapped to CTCs enumeration in CoMMpass (counts/μl). Values were logarithmically transformed, and solid and dashed lines represent median and 25th/75th quantiles, respectively.

**
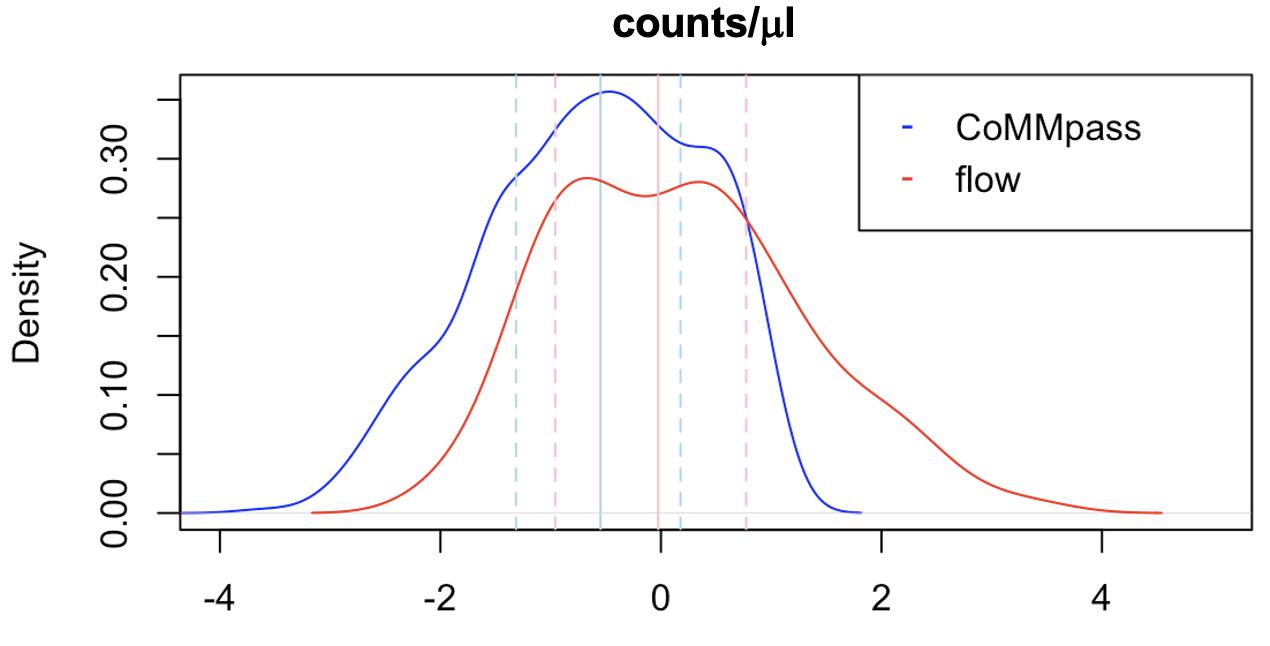
**

**Supplemental Figure 4.** Kaplan-Meier curves for PFS and OS for CoMMpass (**A,B**) and validation dataset (**C,D**). Patients are stratified into logarithmically based groups, depicted by increasing colors. Middle tables show the statistics for pairwise log-rank tests. OS additional comparison refers to patients with ≤10 CTCs or ≤0.001% CTCs vs other groups. ****, p≤0.0001; ***, p≤0.001; **, p≤0.01; *, p≤0.05; ns, non-significant (i.e., p>0.05).

**
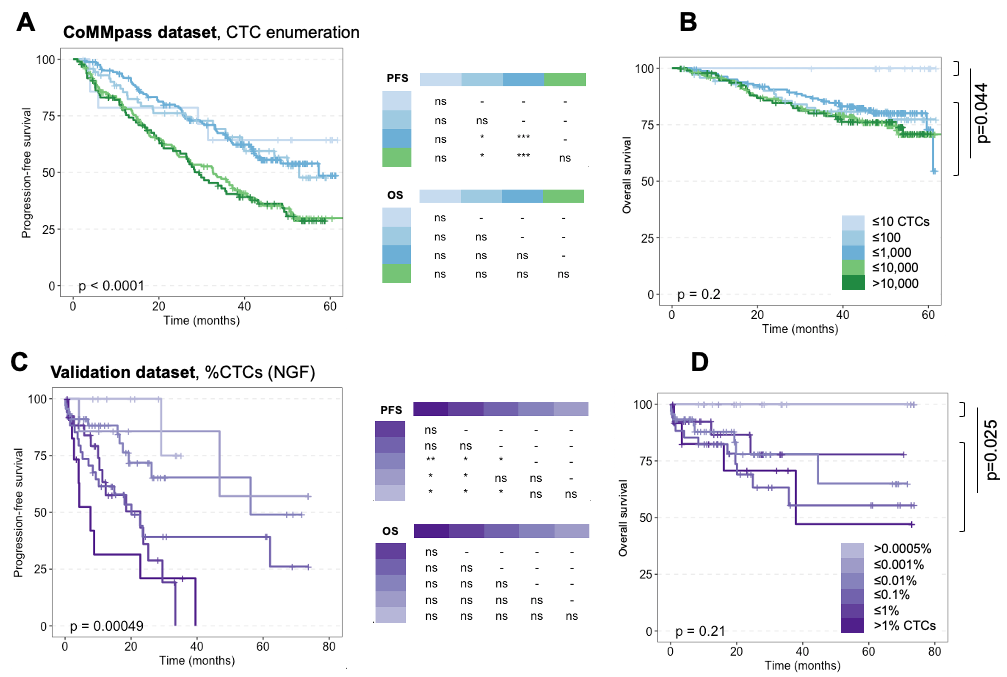
**

**Supplemental Figure 5.** Kaplan-Meier curves for PFS resulting from the combination of CTC levels (≤/> 1,000 CTCs) with other stratifying systems: ISS (**A**), R-ISS (**B**), R2-ISS (**C**), and IMS/IMWG (**D**). C-indexes without and with the incorporation of CTC levels are displayed as barplots.

**
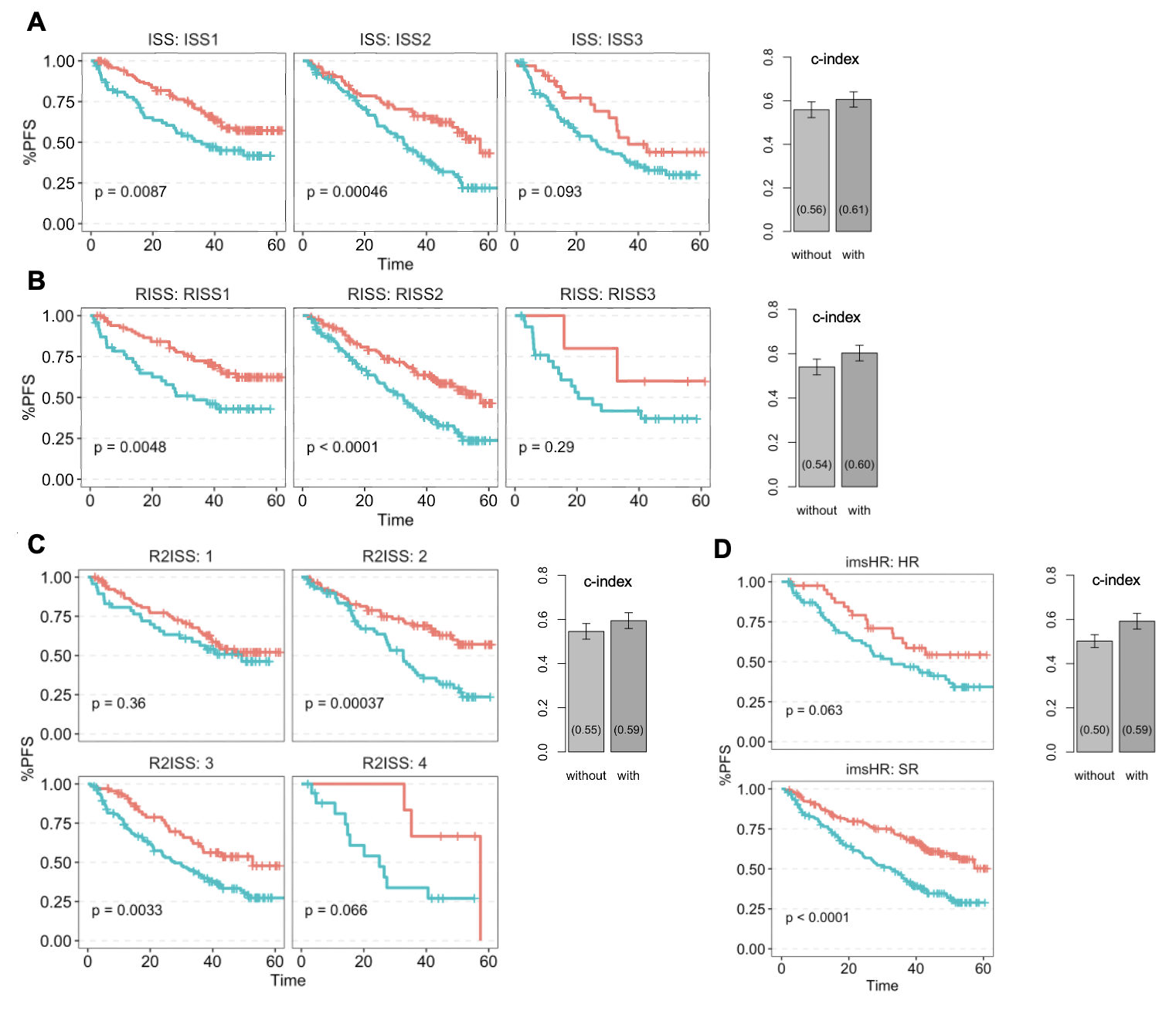
**

**Supplemental Figure 6.** Spearman’s correlations between the number of CTCs (logarithmically transformed) and BM tumor infiltration (**A**), B2-microglobulin (**B**), and M-protein in serum (**C**).


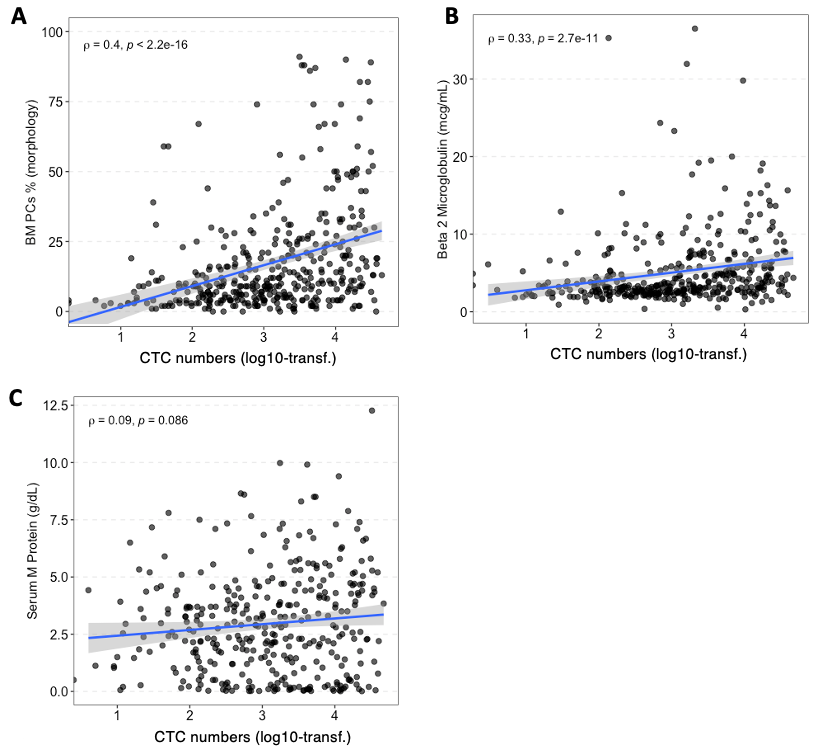


**Supplemental Figure 7.** Supervised cumulative frequency copy number variation plot by chromosome according to the presence of fewer or more than 1,000 CTCs. Gains and losses are colored in red and blue, respectively.

**
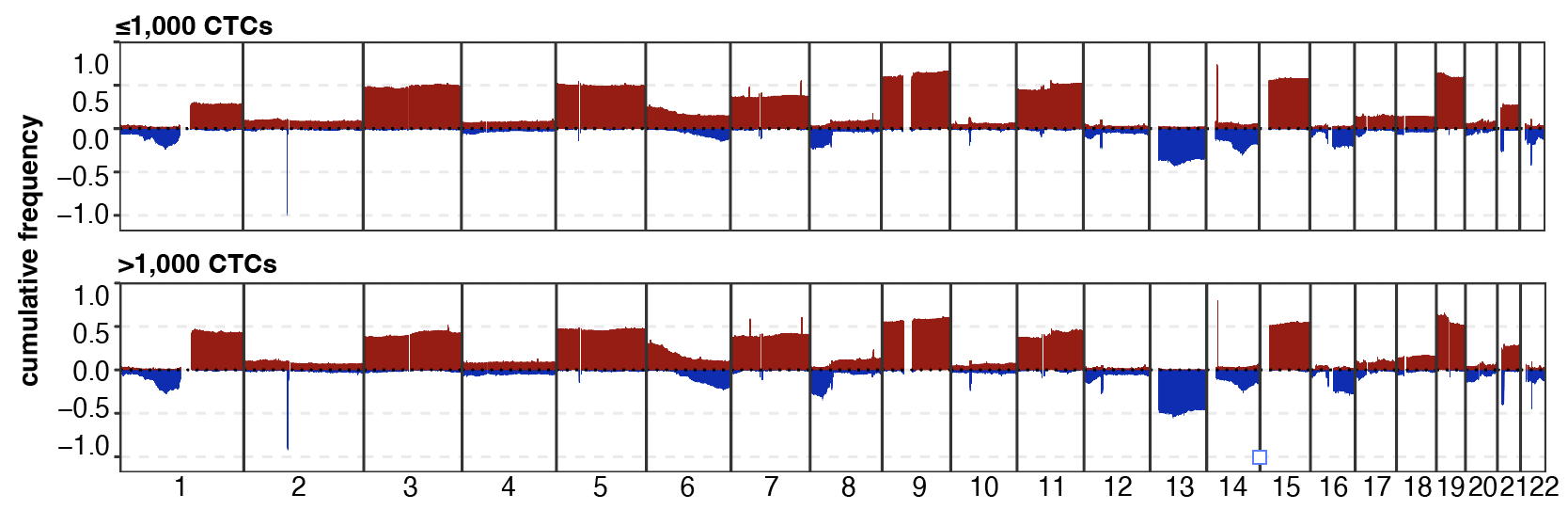
**

**Supplemental Figure 8.** Kaplan-Meier curves for PFS for all prognostically significant IRMMa’s genomic features resulting from the combination with CTC levels. Patients were stratified according to the co-occurrence of high or low CTC levels (i.e., 1,000 CTCs) with WT or mutated events.


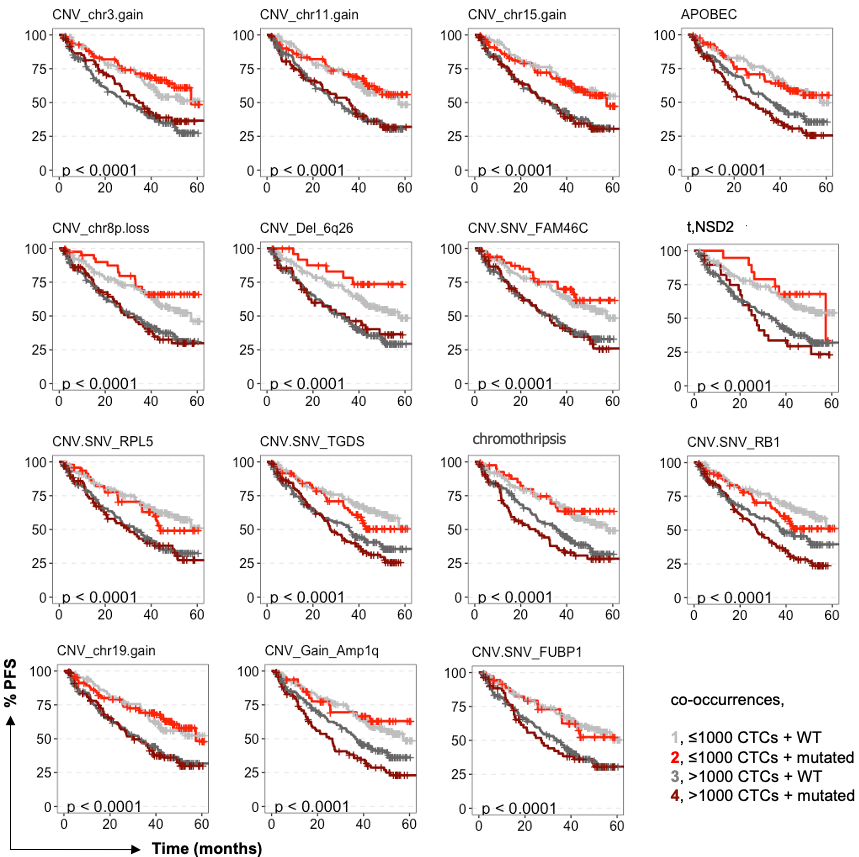


**Supplemental Figure 9.** The same examples of positively (**B**) and negatively (**C**) correlated genes according to CTC numbers (log-10 transformed) for the validation dataset.


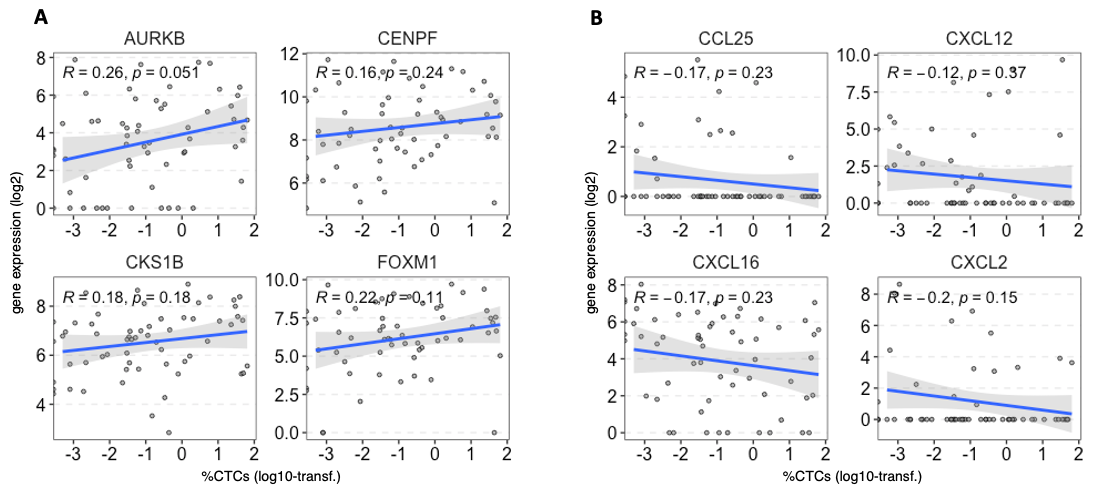


**Supplemental Figure 10.** Genomic regions (purple segments, FDR≤0.01) significantly enriched for differentially expressed genes (red dots) in CoMMpass and the validation dataset.


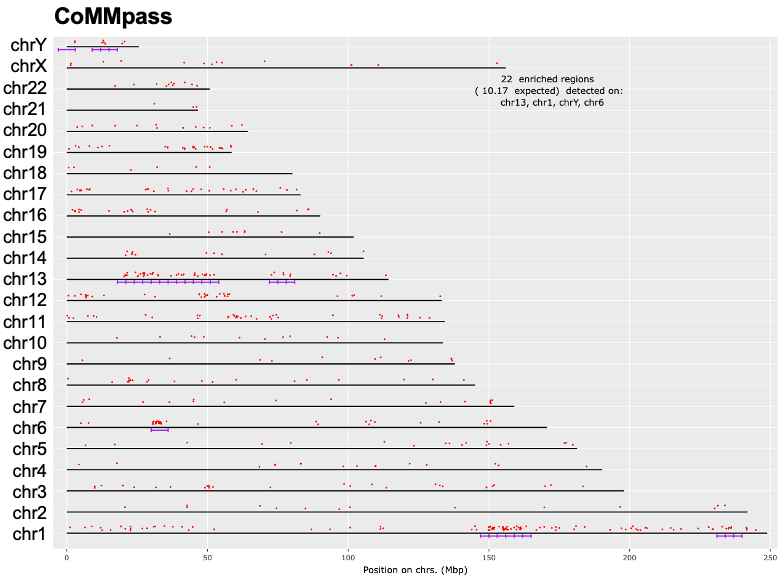


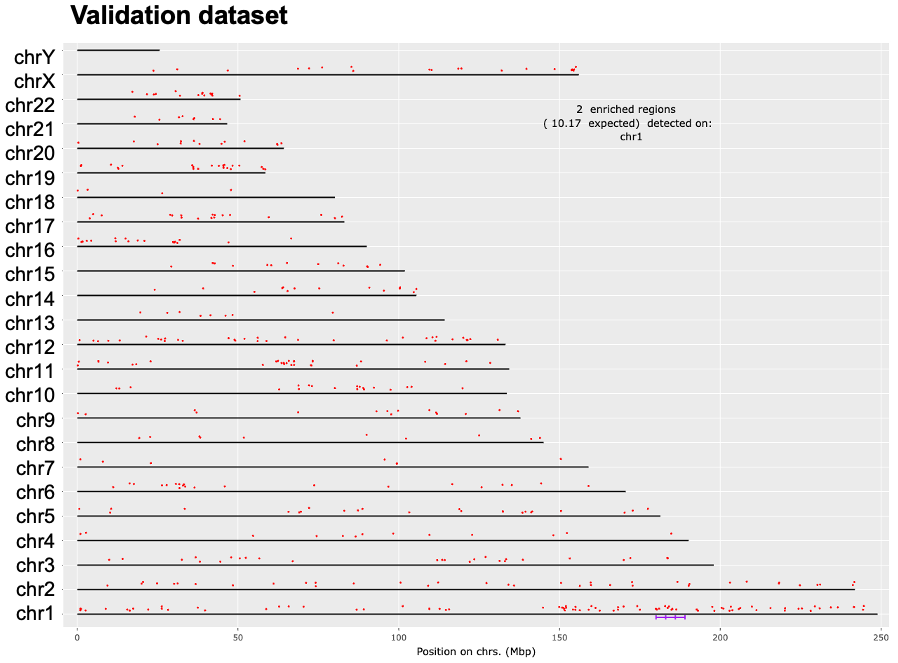


**Supplemental Figure 11.** Correlations between Skerget’s PR signature and CTC levels assessed by CellSearch (**A**) or NGF (**B**), and between the PCL-like score and CTC levels assessed by CellSearch (**A**) or NGF (**D**). Spearman’s correlations are 0.26, 0.33, 0.38, and 0.57, from A to D, respectively.


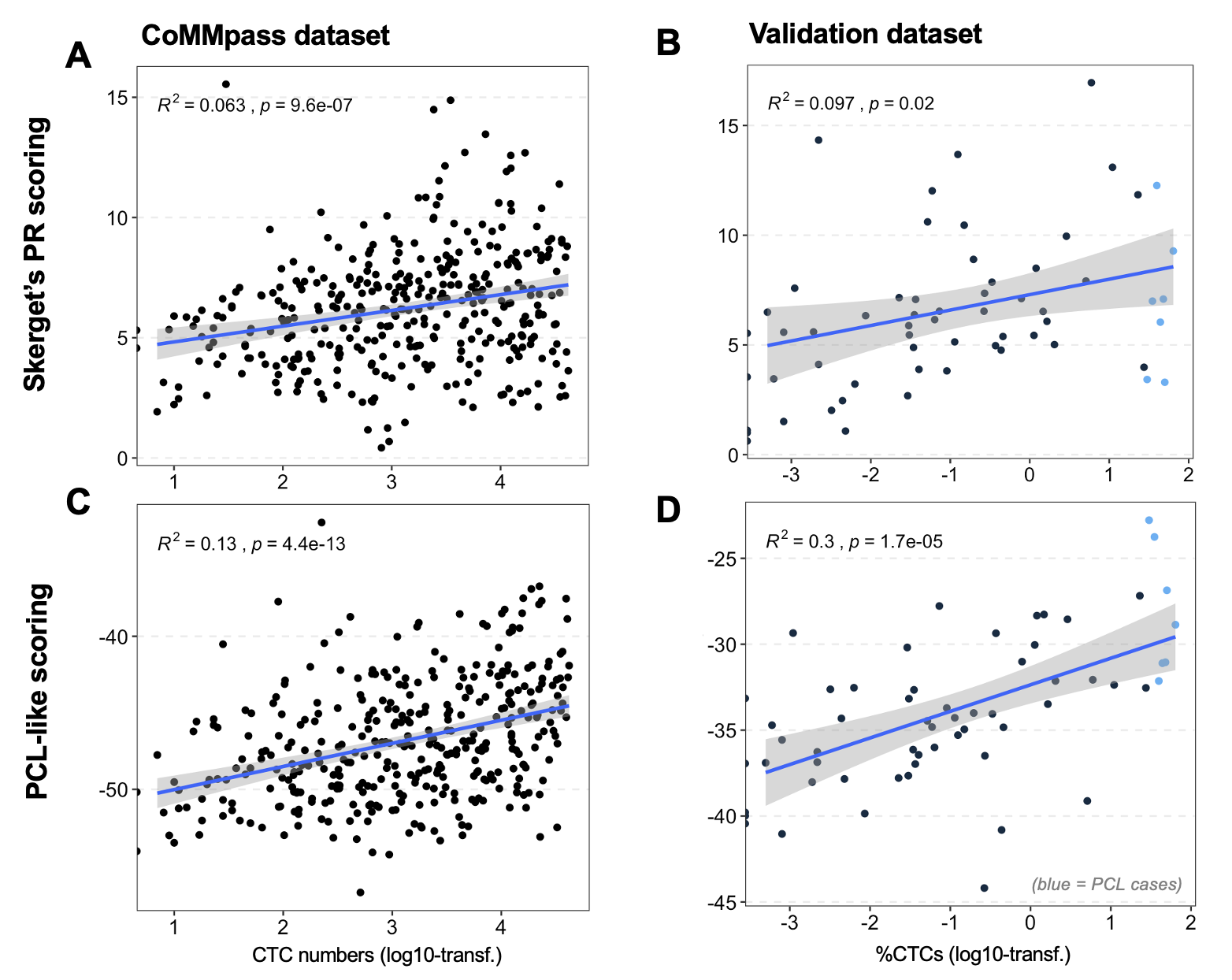


**Supplemental Figure 12.** Kaplan-Meier curves for OS stratified by patients according to PR and CTC levels for CoMMPass (**A**) and validation (**B**) datasets.

**
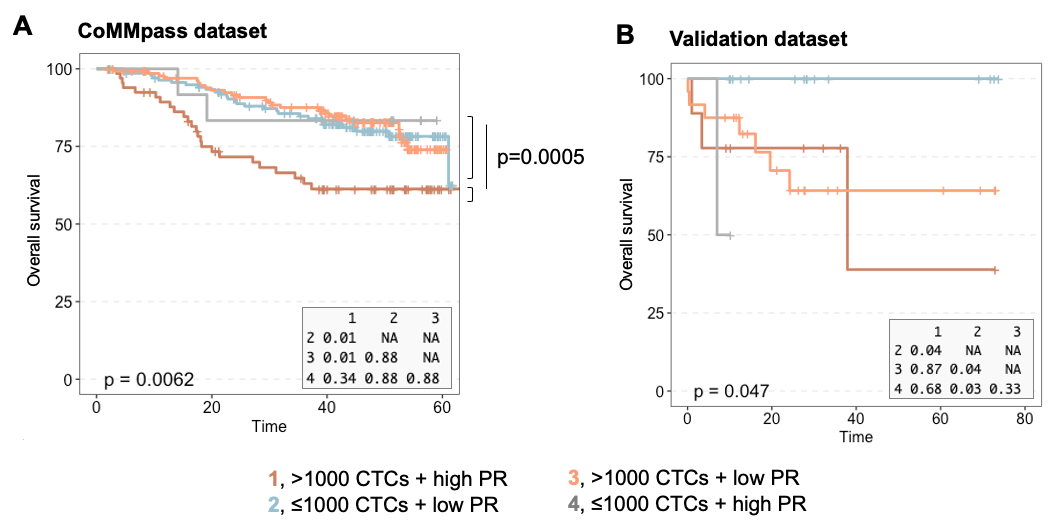
**

**Supplemental Figure 13.** Kaplan-Meier curves for PFS in patients under treatment based on VRD (bortezomib + lenalidomide + dexamethasone; **A**) and the rest of the patients (**B**).

**
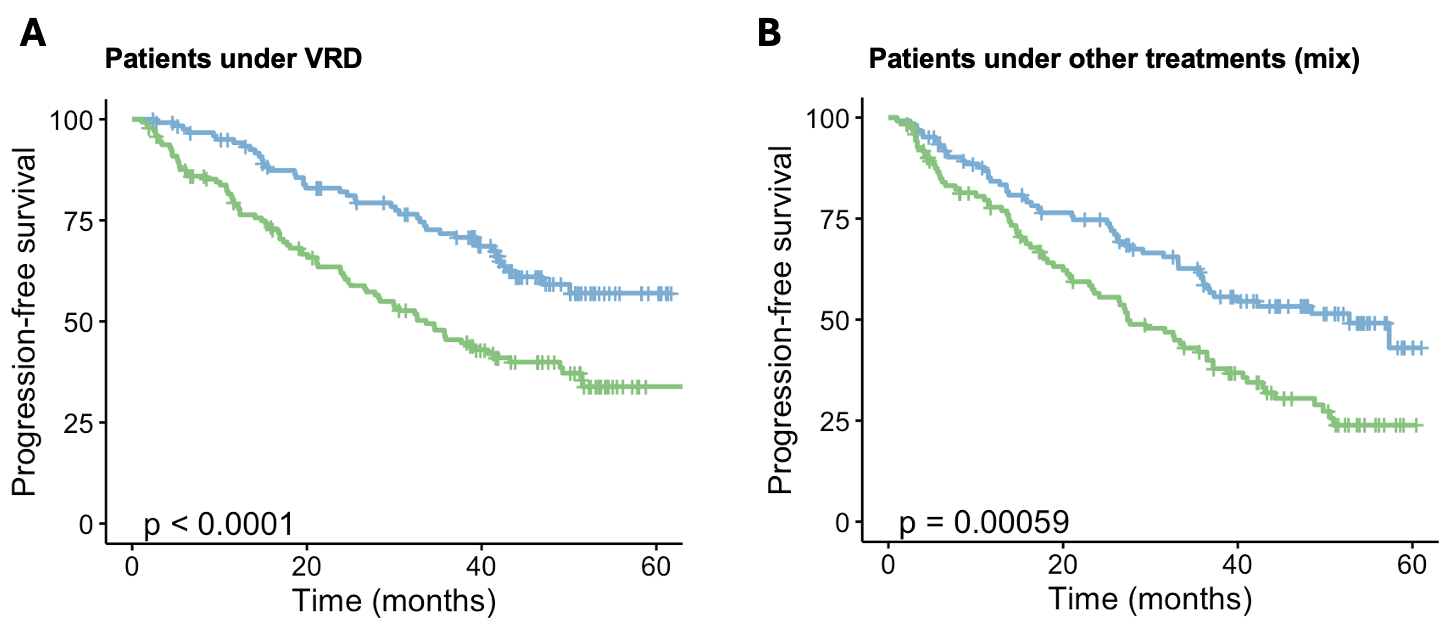
**
