# Supplementary material for: Elevated circulating tumor cells reflect high proliferation and genomic complexity in multiple myeloma
Source: Hemasphere. 2025 Sep 23;9(9):e70218. doi: 10.1002/hem3.70218 (PMC12455875; doi:10.1002/hem3.70218)
Supplement: Supplementary file 2 — Supporting Information. [file HEM3-9-e70218-s004.docx]

**Supplemental Material and Methods for:**

**Elevated circulating tumor cells reflect high proliferation and genomic complexity in multiple myeloma**

**Patients and samples**

*Patient cohorts.* The Multiple Myeloma Research Foundation (MMRF) coordinated the Relating Clinical Outcomes in Multiple Myeloma to Personal Assessment of Genetic Profile (CoMMpass) study (NCT01454297), where they collected longitudinal clinical and sequencing information of 1,154 multiple myeloma (MM) patients from the United States, Canada, Spain, and Italy between 2011 and 2023. Of these patients, information on circulating tumor cells (CTCs) was available in 540 patients at baseline.

The validation dataset from the University Hospital Ostrava (Czech Republic) contains baseline clinical information on a total of 135 NDMM patients diagnosed between 2018 and 2025. Patients with ≥20% CTCs, considered as primary plasma cell leukemia (PCL), were excluded from the overall analysis to ensure reliable comparison with the training cohort. However, some patients with primary PCL were included in downstream genomic and transcriptomic analyses; see the respective sections for experimental design details. All patients were treated with the routine standard of care triplet regimens consisting of proteasome inhibitors, immunomodulatory drugs, and corticosteroids, including autologous stem cell transplantation in eligible patients below 70-75 years old. CTC information at baseline was available for all patients. The study was approved by the Ethics Committee of the University Hospital Ostrava (approval number 433/2012) and conducted in accordance with the Declaration of Helsinki.

*CTCs assessment.* For the CoMMpass dataset, CTC assessments were performed using the *CellSearch System* (Menarini Silicon Biosystems) as previously described.^1^ Briefly, peripheral blood (PB) was collected in CellSave tubes and then magnetically sorted by CD138+ beads. Next, they were stained with a color panel including CD38, CD45, CD19, and DAPI, and enumerated by trained operators through the *BioMarQ* software (Menarini Silicon Biosystems). CTCs were defined as events with rounded to oval morphology, positive for DAPI and CD38, and negative for CD45 and CD19. Results are expressed as the number of cells/4 ml of PB and were logarithmically transformed (base 10) to normalize their skewed distribution.

For the validation dataset, CTCs were evaluated through next-generation flow (NGF) cytometry according to EuroFlow’s standard operating protocols.^2^ Prior to CTC analysis, matched BM samples were assessed to determine the phenotype of clonal plasma cells (PC) using the complete EuroFlow plasma cell dyscrasia (PCD) panel. For CTC analysis, PB samples were collected in EDTA-anticoagulated tubes and processed within 24 hours according to the bulk lysis protocol for MRD processing. Samples were stained using the eight-color PCD Tube 1, which included the following fluorochrome-antibody conjugates: CD38 multi-epitope FITC (clone “multi epitope,” Cytognos, Salamanca, Spain), CD56 PE (C5.9, Cytognos), CD45 PerCP-Cy5.5 (HI30, Exbio, Prague, Czech Republic), CD19 PE-Cy7 (J3-119, Beckman Coulter, Brea, CA, USA), CD117 APC (104D2, BD Biosciences, San Jose, CA, USA), CD81 APC-C750 (M38, Cytognos), CD138 BV421 (MI15, BD Biosciences), and CD27 BV510 (O323, BioLegend, San Diego, CA, USA). Data acquisition was performed using a FACS Canto II flow cytometer (BD Biosciences). CTCs were identified based on the aberrant phenotype determined from the corresponding BM sample, and data analysis was conducted using *Infinicyt* software (version 2.0; BD Biosciences). CTCs were enumerated as a percentage of total CD45+ PB leukocytes. A CTC cluster consisting of >20 events was considered a valid threshold with a limit of detection (LOD) defined according to the following formula. Median LOD of NGF in the validation cohort was 0.0005%.

*LOD = 20 / (Leukocyte count) × 100*

*Cytogenetic evaluation.* FISH analysis was performed on immunomagnetically enriched CD138+ PCs in Ostrava’s validation dataset using the following probes: MetaSystems XL RB1/DLEU/LAMP; XL IGH plus; XL P53; XL 1p32/1q21; XL 5p15/9q22/15q22 (hyperdiploidy); XL t(11;14); XL t(4;14); and XL t(14;16). A total of 100 nuclei were evaluated. Hybridization signals were analyzed using a fluorescence microscope (Olympus BX41). The CoMMpass dataset relied on sequencing data to estimate these aberrations (i.e., seq-FISH).^3^

*Statistical analyses.* Progression-free (PFS) was defined as the time from patient diagnosis to disease progression or death from any cause, and overall survival (OS) as the time from diagnosis to death from any cause. Both were studied through Kaplan-Meier curves and the log-rank test. Kruskal-Wallis’ and its post-hoc test (i.e., Dunn’s tests) were respectively used for 2-based or 2+ comparisons when checking CTCs’ distribution according to clinical variables, stratifying systems, and IRMMa’s genomic groups. Correlation coefficients were based on Spearman’s method. Adjusted P-values are based on Benjamini-Hochberg’s correction for all subgroups in each clinical/cytogenetic variable. Early-progressing patients were defined as those progressing before or at 18 months of treatment.

The CTC-based cutoff (i.e., median) was additionally confirmed through a bootstrapped version of the *surv_cutpoint* function from the *survminer* package.^4^  It was based on PFS times and configured to keep at least a representation of 20% in each split, then bootstrapped 1,000 to repetitions. The final cutoff was 963 CTCs.

**Whole-genome and whole-exome sequencing analysis**

*Sample sequencing and preprocessing.* Whole-genome and whole-exome sequencing (WGS/WES) data included in the CoMMpass dataset used in this study comprise a total of 460 patients. Full processing and data generation have been previously described;^1^ this study uses BM-derived information. BM samples were magnetically sorted based on CD138+ expression to study tumoral fractions, while keeping CD138- mononuclear cells as normal controls. Sequencing was aligned against the GRCh37 genome build. Single-nucleotide variants (SNVs) and short insertion-deletion variants (INDELs) were called by using a pipeline based on *Mutect2* and *Strelka*.^5^  Copy number variants (CNVs) were called using *tCoNuT* (github.com/tgen/tCoNuT), with MMRF CoMMpass-specific optimizations. Structural variants (SVs) were obtained via consensus calling of *Manta* and *delly*,^6,7^ followed by manual curation, as previously described.^8^

For the validation cohort, a total of 12 samples were selected to reflect the extreme values of the CTC logarithmic ranges described above: undetectable CTCs (n=3), ≥0.0005% (n=2), ≥1% (n=4), and ≥10% CTCs (n=3, all cases with PCL). Then, BM samples were FACS-sorted according to the same aberrant phenotype detected in the CTC assessment, with PB mononuclear cells utilized as normal controls. Pathological BM PCs and DNA isolation were performed as described in the RNA-seq section. DNA concentration was assessed using the Qubit 2.0 Fluorometer (Thermo Fisher Scientific, USA). WGS libraries were prepared from 2-2.5 ng of DNA using the NEBNext® Ultra™ II FS DNA Library Prep Kit for Illumina (New England Biolabs, USA), according to the manufacturer’s instructions. DNA was enzymatically fragmented for 15 minutes, libraries were amplified using eight cycles of PCR, and final libraries were assessed for quantity and quality by Qubit 2.0 Fluorometer and Agilent TapeStation 2200 (Agilent Technologies, USA). Libraries were pooled in equimolar ratios and sequenced on an Illumina NovaSeq X Plus platform with a 25B flow cell at the New York Genome Center (USA). Samples were sequenced at a median coverage of 52X (range: 42-82X) for tumor samples and 37X (range: 28-45X) for normal samples.

Sequencing data were processed using the *Sarek* pipeline,^9^ using the GRCh38 reference genome. SNVs and INDELs were called using *Freebayes*,^10^ *Mutect2*,^11^ and *Strelka^12^* in a two-caller consensus approach.^5^  Additional filtering steps were applied to exclude likely artifacts, including variants with strand bias (Fisher’s Exact Test), low variant allele frequency (VAF < 0.05), and mutant allele read count below four. Tumor purity, ploidy, and CNV) were inferred using *ASCAT*.^13^ SVs were identified with *Manta*,^6^ and chromothripsis was manually annotated as previously described.^8^ Definitions of the different APOBEC mutagenesis status are (i) hyper-APOBEC, as having APOBEC contribution in the top 10th decile when pooling WGS data with 361 newly diagnosed MM patients from a previous publication;^14^ (ii) APOBEC for all the others between deciles 1-10, as previously described;^15^ and (iii) wild-type (WT) when the APOBEC contribution was below the 1st decile.

*Genomic Feature Definition and Genomic Classification.* Prognostically relevant genomic features were selected from the previously published IRMMa prediction model.^16^ The model can be applied to WGS data with inputs inclusive of CNVs, SNVs/indels, mutational signatures, and canonical translocations (i.e., FISH and/or WGS data) in MM. The code to generate a feature table and genomic classification for one of 12 molecular subgroups is available at <https://github.com/UM-Myeloma-Genomics/GCP_MM>. Genomic feature tables and molecular classification were likewise performed for the validation cohort, with modifications to the code to allow for GRCh38. “CNV/SNV” denotes a composite of mutational and deletional inactivation for punctual genes (e.g., *CNV.SNV_FAM46C*).

*Bioinformatic analysis.* For the discovery cohort (CoMMpass), high and low CTC counts were defined as CTC counts above and below 1,000 CTCs, respectively. After removing genomic features present in less than 5 cases (19/132), each one was tested for enrichment in high or low CTC subgroups via Kruskal or Dunn’s test when single or multi-hit events, respectively; significance was considered below (unadjusted) p≤0.05.

To assess if other recurrent genomic features not included in the IRMMa model were associated with CTC count, we assessed the representation of 68 previously identified SV hotspots.^8^ All SVs were intersected with an allowance for SVs within 1MB of the hotspot limits. Manual curation was performed to select high-confidence SV events as matches between amplification hotspots and CNV gains/supported SV, while deletion hotspots were matched with CNV losses and supported SV. Using a Wilcoxon Rank Sum test, the frequency of high-confidence SV events was tested between high and low CTC subgroups. Where available, RNAseq data were interrogated to assess the effect of SV events on target gene expression.

For survival analyses, the mutational status of IRMMa’s genomic features was combined with the presence of more or fewer than 1,000 CTCs and tested using Kaplan-Meier or Cox regression models to obtain the survival probabilities or for subgroup analyses, respectively. Features or genomic groups with few cases with survival information (n≤5) or “infinite” lower/upper hazard-ratio values were discarded. Significance was considered with (unadjusted) p≤0.2. P-values were adjusted according to Benjamini-Hochberg’s correction.

**RNA-sequencing analysis**

*Sample sequencing and preprocessing.* For the CoMMPass dataset, CD138+ sorted BM tumor cells were processed as previously described.^1^ RNA-sequencing (RNAseq) analyses were based on version IA22, using gene-level count estimated counts calculated through *Salmon*. Samples were filtered out if (i) RIN values <6, (ii) a percentage of mitochondrial genes ≥10%, or (iii) a percentage of ribosomal genes ≥1%. Then, we removed patients with no CTC or BM PC infiltration information, leaving a final number of 279 cases.

For the validation dataset, a total of 60 patients were analyzed. Samples were selected to represent all the logarithmic groups defined by CTC levels: undetectable CTCs (n=5), ≥0.0005% (n=4), ≥0.001% (n=9), ≥0.01% (n=13), ≥0.1% (n=11), ≥1% (n=8), and ≥10% CTCs (n=10), This dataset included nine samples with CTC levels over 20%, formally meeting the traditional criteria for PCL diagnosis. Samples were obtained from the Biobank in the form of frozen BM mononuclear cells or PCs isolated by MACS using CD138 magnetic beads (Miltenyi Biotec, Germany). PCs from BM mononuclear samples were then FACS-sorted into RPMI-1640 medium supplemented with 10% fetal bovine serum. Sorting was performed based on the original BM aberrant immunophenotype using a BD FACSAria III instrument (BD Biosciences, USA). Sorted PCs were processed using the AllPrep DNA/RNA Micro Kit (Qiagen, Germany). RNA quantity and quality were assessed using a Qubit 2.0 Fluorometer (Thermo Fisher Scientific, USA) and an Agilent TapeStation 2200 (Agilent Technologies, USA), respectively. RNA-sequencing libraries were prepared from total RNA (2-3.5 ng) using the SMARTer Stranded Total RNA-Seq Kit v2 (Takara Bio, Japan), following the manufacturer’s protocol. Libraries were amplified using 14 cycles of PCR, and their quantity and quality were evaluated using the Qubit 2.0 Fluorometer and Agilent TapeStation 2200. Finally, libraries were pooled in equimolar ratios and sequenced on a NovaSeq X Plus platform (Illumina Inc., San Diego, USA) at Macrogen Europe (The Netherlands). A median sequencing depth of 38.25 million reads per sample (range: 16.04-60.99 million reads) was obtained across all 60 samples.

*Bioinformatic analyses.* Further filtering steps involved removing genes with less than 10 reads in at least 40% of the samples (20% in the validation dataset) and all immunoglobulin genes. Potential batch effects were investigated, but no correction was applied as no strong confounding factors were found. Packages *EdgeR^17^* and *limma^18^* were used to build a linear model considering the logarithmic-transformed value of CTC counts and gene expression levels. The linear model was corrected to include the global effect of the BM tumor burden on CTC numbers.

*y = β_0_​ + β_1_X + β_2_B,* where

*y* corresponds to each gene expression level,

*X* corresponds to log10(CTCs + ∂), where ∂ equals 0.1 or 0.0001 for CoMMpass and the validation dataset, respectively, and

*B* corresponds to BM infiltration (i.e., *D_IM_MORPHOLOGY_PERCENT_PC_IN_BM*).

For subsequent analyses, only differentially expressed genes (DEGs) with a Benjamini-Hochberg (BH)-corrected FDR ≤0.01 or ≤0.1 were selected for the discovery and validation datasets, respectively. These DEGs were then annotated using *EnsDb.Hsapiens.v75*. In parallel, another linear modeling based on the *stats* package and considering the same formula was used to obtain Pearson’s correlation coefficients. These results were later merged with previous DEGs.

Gene-set enrichment analysis (GSEA) was performed by using *clusterProfiler* considering the biological process (BP) ontology (with default parameters except for *eps*=0, *pvalueCutoff*=1, and *nPermSimple*=10000);^19^ significant DEGs were ordered according to their Pearson’s coefficient. The top 10 positive and negative results were plotted together. ShinyGO^20^ was used to see chromosomal regions enrichment according to significant DEGs, adjusting the FDR cutoff for windows to 0.01.

Previously published proliferation (PR) and PCL-like gene signatures^1,21,22^ were transferred to our datasets by multiplying the gene expressions by the described coefficients and summing them together. These were later correlated (Spearman’s coefficient) with CTC counts. To define prognostic groups, we considered values above the third quantile as a high PR value and above 1,000 as elevated CTC counts.

**REFERENCES**

1. Skerget S, Penaherrera D, Chari A, et al. Comprehensive molecular profiling of multiple myeloma identifies refined copy number and expression subtypes. Nature Genetics 2024;56(9):1878-1889, doi:10.1038/s41588-024-01853-0

2. Jelinek T, Bezdekova R, Zihala D, et al. More Than 2% of Circulating Tumor Plasma Cells Defines Plasma Cell Leukemia–Like Multiple Myeloma. Journal of Clinical Oncology 2023;41(7):1383-1392, doi:10.1200/jco.22.01226

3. Miller C, Yesil J, Derome M, et al. A Comparison of Clinical FISH and Sequencing Based FISH Estimates in Multiple Myeloma: An Mmrf Commpass Analysis. Blood 2016;128(22):374, doi:10.1182/blood.v128.22.374.374

4. Kassambara A, Kosinski M, Biecek P: survminer: Drawing Survival Curves using ‘ggplot2,’ 2020.

5. Benjamin D, Sato T, Cibulskis K, et al. Calling Somatic SNVs and Indels with Mutect2. bioRxiv 2019;861054, doi:10.1101/861054

6. Chen X, Schulz-Trieglaff O, Shaw R, et al. Manta: rapid detection of structural variants and indels for germline and cancer sequencing applications. Bioinformatics 2015;32(8):1220-1222, doi:10.1093/bioinformatics/btv710

7. Rausch T, Zichner T, Schlattl A, et al. DELLY: structural variant discovery by integrated paired-end and split-read analysis. Bioinformatics 2012;28(18):i333-i339, doi:10.1093/bioinformatics/bts378

8. Rustad EH, Yellapantula VD, Glodzik D, et al. Revealing the impact of structural variants in multiple myeloma. Blood Cancer Discovery 2020;1(3):bloodcandisc.0132.2020, doi:10.1158/2643-3230.bcd-20-0132

9. Garcia M, Juhos S, Larsson M, et al. Sarek: A portable workflow for whole-genome sequencing analysis of germline and somatic variants. F1000Research 2020;9(63, doi:10.12688/f1000research.16665.2

10. Garrison E, Marth G. Haplotype-based variant detection from short-read sequencing. arXiv 2012, doi:10.48550/arxiv.1207.3907

11. McKenna A, Hanna M, Banks E, et al. The Genome Analysis Toolkit: A MapReduce framework for analyzing next-generation DNA sequencing data. Genome Research 2010;20(9):1297-1303, doi:10.1101/gr.107524.110

12. Kim S, Scheffler K, Halpern AL, et al. Strelka2: fast and accurate calling of germline and somatic variants. Nature Methods 2018;15(8):591-594, doi:10.1038/s41592-018-0051-x

13. Ross EM, Haase K, Loo PV, et al. Allele-specific multi-sample copy number segmentation in ASCAT. Bioinformatics 2021;37(13):1909-1911, doi:10.1093/bioinformatics/btaa538

14. Cirrincione AM, Poos AM, Ziccheddu B, et al. The biological and clinical impact of deletions before and after large chromosomal gains in multiple myeloma. Blood 2024, doi:10.1182/blood.2024024299

15. Maura F, Rajanna AR, Ziccheddu B, et al. Genomic Classification and Individualized Prognosis in Multiple Myeloma. Journal of Clinical Oncology 2024;JCO2301277, doi:10.1200/jco.23.01277

16. Bolli N, Biancon G, Moarii M, et al. Analysis of the genomic landscape of multiple myeloma highlights novel prognostic markers and disease subgroups. Leukemia 2018;32(12):2604-2616, doi:10.1038/s41375-018-0037-9

17. Robinson MD, McCarthy DJ, Smyth GK. edgeR: a Bioconductor package for differential expression analysis of digital gene expression data. Bioinformatics 2009;26(1):139-140, doi:10.1093/bioinformatics/btp616

18. Ritchie ME, Phipson B, Wu D, et al. limma powers differential expression analyses for RNA-sequencing and microarray studies. Nucleic Acids Research 2015;43(7):e47-e47, doi:10.1093/nar/gkv007

19. Yu G, Wang L-G, Han Y, et al. clusterProfiler: an R Package for Comparing Biological Themes Among Gene Clusters. OMICS: A Journal of Integrative Biology 2012;16(5):284-287, doi:10.1089/omi.2011.0118

20. Ge SX, Jung D, Yao R. ShinyGO: a graphical gene-set enrichment tool for animals and plants. Bioinformatics 2019;36(8):2628-2629, doi:10.1093/bioinformatics/btz931

21. Zhan F, Huang Y, Colla S, et al. The molecular classification of multiple myeloma. Blood 2006;108(6):2020-2028, doi:10.1182/blood-2005-11-013458

22. Bruinink DHo, Kuiper R, Duin Mv, et al. Identification of High-Risk Multiple Myeloma With a Plasma Cell Leukemia-Like Transcriptomic Profile. Journal of Clinical Oncology 2022;JCO2101217, doi:10.1200/jco.21.01217
