# Supplementary material for: Elevated circulating tumor cells reflect high proliferation and genomic complexity in multiple myeloma
Source: Hemasphere. 2025 Sep 23;9(9):e70218. doi: 10.1002/hem3.70218 (PMC12455875; doi:10.1002/hem3.70218)
Supplement: Supplementary file 3 — Supporting Information. [file HEM3-9-e70218-s001.docx]

**Supplemental Tables for:**

**Elevated circulating tumor cells reflect high proliferation and genomic complexity in multiple myeloma**

| **INDEX** |  |
| --- | --- |
| **Table 1. Clinical features of CoMMpass and validation dataset.** | **2** |
| **Table 2. CTC association with clinical and stratifying markers.** | **[excel]** |
| **Table 3. CTC association with cytogenetic aberrations.** | **[excel]** |
| **Table 4. CTC and genomic features association.** | **[excel]** |
| **Table 5. Multivariable analysis with IRMMa’s features** | **[excel]** |
| **Table 6. Prognostic value of combining CTCs and genomic groups/features.** | **[excel]** |
| **Tables 7 and 8. Differentially expressed genes (linear model), both datasets.** | **[excel]** |
| **Tables 9 and 10. GSEA analyses, both datasets.** | **[excel]** |

# **Supplemental Table 1.** Clinical characteristics of patients included in the study. Discovery (CoMMpass) and validation datasets (Czech Republic).

|  | **CoMMpass (n = 540)** | **Czech Rep. (n = 135)** |
| --- | --- | --- |
| **Age (median, range), years** | 63 (27 - 93) | 68 (40 - 87) |
| **Sex (n, %)** |  |  |
| Female | 202 (37.4%) | 61 (45.2%) |
| Male | 316 (58.5%) | 74 (54.8%) |
| **ISS** |  |  |
| I | 191 (35.4%) | 41 (30.4%) |
| II | 182 (33.7%) | 44 (32.6%) |
| III | 126 (23.3%) | 47 (34.8%) |
| **R-ISS** |  |  |
| I | 133 (24.6%) | 30 (22.2%) |
| II | 312 (57.8%) | 60 (44.4%) |
| III | 36 (6.7%) | 20 (14.8%) |
| **R2-ISS** |  |  |
| I | 163 (30.2%) | 15 (11.1%) |
| II | 153 (28.3%) | 27 (20%) |
| III | 200 (37%) | 54 (40%) |
| IV | 24 (4.4%) | 20 (14.8%) |
| **LDH levels** |  |  |
| Normal | 378 (70%) | 102 (75.6%) |
| High | 50 (9.3%) | 31 (23%) |
| **Cytogenetic risk*** |  |  |
| SR | 434 (80.4%) | 107 (79.3%) |
| HR | 106 (19.6%) | 28 (20.7%) |
| **Classical cytogenetic aberrations** |  |  |
| del17p | 49 (9.1%) | 12 (8.9%) |
| t(4;14) | 52 (9.6%) | 16 (11.9%) |
| t(14;16) / t(14;20) | 17 (3.1%) | 2 (1.5%) |
| **New cytogenetic aberrations** |  |  |
| gain1q | 122 (22.6%) | 42 (31.1%) |
| amp1q | 28 (5.2%) | 20 (14.8%) |
| del1p | 57 (10.6%) | 9 (6.7%) |
| del13q | 207 (38.3%) | 54 (40%) |
| **BM infiltration (morphology)** | 10 (0 - 91) | 19.6 (0.4 - 93.6) |
| **CTC levels (median, range)** | 1,132 (0 - 47,146) CTCs | 0.0209 (0 - 11)% CTCs |
| *High-risk cytogenetics defined as del(17p), t(4;14), and/or t(14;16). | | |
